# Preparation of Electrode Materials Based on Carbon Cloth via Hydrothermal Method and Their Application in Supercapacitors

**DOI:** 10.3390/ma14237148

**Published:** 2021-11-24

**Authors:** Xiaonan Wang, Peiquan Xu, Pengyu Zhang, Shuyue Ma

**Affiliations:** 1 College of Materials Engineering, Shanghai University of Engineering Science, Shanghai 201620, China; 15738773508@163.com (X.W.); pengyu9711@163.com (P.Z.); mashuyue1998@163.com (S.M.); 2 Shanghai Collaborative Innovation Center of Laser Advanced Manufacturing Technology, Shanghai 201620, China

**Keywords:** supercapacitors, carbon materials, conductive polymers, metal compounds, composite materials, carbon cloth

## Abstract

Supercapacitors have the unique advantages of high power density, fast charge and discharge rates, long cycle life, high safety, and reliability, and are increasingly being used for applications including automobiles, rail transit, communication equipment, digital electronics, and aerospace equipment. The supercapacitor industry is currently in a stage of rapid development; great breakthroughs have also been made in improving the performance of supercapacitors and the expansion of their application. Electrode technology is the core of supercapacitors. Transition-metal compounds have a relatively high theoretical capacity and have received widespread attention as electrode materials for supercapacitors. In addition, there is a synergistic effect between the different components of various electrode composite materials. Due to their superior electrochemical performance, supercapacitors are receiving increasing research attention. Flexible supercapacitors have been hailed for their good plasticity, resulting in a development boom. This review article mainly outlines the development process of various electrode materials, including carbon materials, conductive polymers, metal compounds, and composite materials, as well as flexible electrode materials based on carbon cloth.

## 1. Introduction

Recently, the explosive growth of society and constant advancement of science and technology have led to an increasing demand for energy, resulting in a significant energy crisis and environmental problems [1,2]. There is thus an urgent need to develop environmentally friendly, efficient, clean, and sustainable advanced energy conversion and storage technologies [3,4]. Currently, the development of the new energy industry is based on the three core products of secondary batteries (mainly lithium-ion batteries) [5], supercapacitors [6], and fuel cells [7], with a vast application network.

Supercapacitors are also known as large-capacity capacitors, energy storage capacitors, gold capacitors, electric double layer capacitors, and farad capacitors. After decades of development, using carbon material from naturally abundant lignocellulose biomass precursors as the electrode material of flexible/wearable electronic devices is a future trend, but there are still technological challenges [8]. Based on availability and low-cost, various carbon materials, such as activated carbons (AC), nitrogen doping on carbon, and graphene on carbon cloth, have also been studied [9,10]. However, their performance is not particularly good.

Initially, in 1957, Becker filed an application to use high-specific-surface-area activated carbon as the electrode material for supercapacitors. In 1962, the Standard Oil Company commercialized carbon material electrochemical capacitors. In 1979, the Nippon Electronic Company (NEC) implemented supercapacitors for large-scale commercial applications. Scholars are increasingly discovering the application value of supercapacitors. The structure of a supercapacitor consists of the electrodes, electrolyte, and separator. The electrode material in the electrodes plays a vital role in the performance of the supercapacitor [11]. The electrode material is the core. From carbon materials [12,13], element-doped carbon materials [14,15], metal compounds [16,17], multi-metal compounds [18,19], and polymers [20,21], to various composite materials, various superior porous three-dimensionally structured composite materials have been constructed. With the further development of society, there has been a greater pursuit of wearable and strong plasticity flexible electrodes. The preparation of carbon cloth offers the advantages of low cost, good flexibility, and good conductivity, and, as a substrate, has been hailed as a high-performance electrode material [22,23,24,25,26].

**Figure 1:**
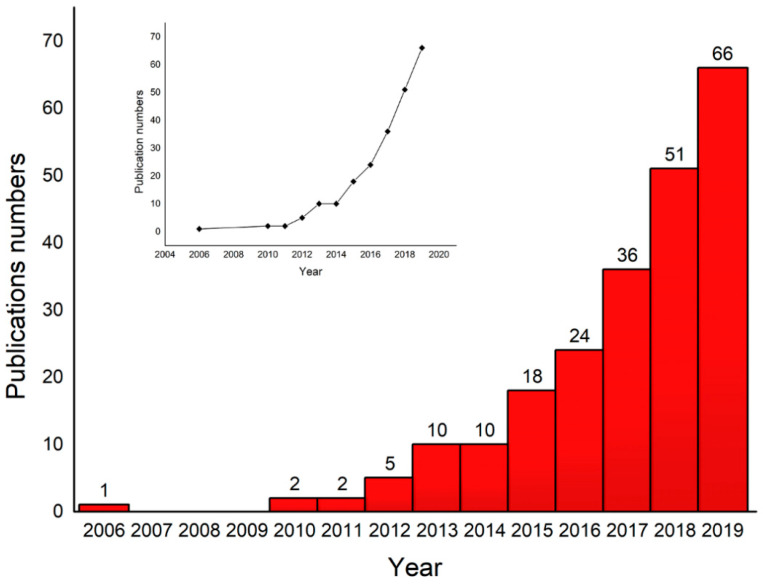
Increasing trend of the number of publications related to supercapacitors based on CC in the past decade. These results were obtained by searching the Web of Science for articles with the keywords “supercapacitor”, “carbon cloth”, and “carbon fiber cloth” included in their titles.

CC has been used as an electrode material for flexible supercapacitors applied in portable and wearable products, setting off a research boom. Figure 1 clearly shows that in the past ten years, there has been a linear upward trend in the number of publications with the keywords “supercapacitor”, “carbon cloth”, and “carbon fiber cloth” included in their titles (from a search in Web of Science), and this trend is expected to be maintained. Flexible supercapacitors based on CC are very promising electrode materials. CC has the advantages of light weight, low cost, excellent flexibility, large surface area, high conductivity, and porosity; in addition, it is particularly easy to construct an electrode material with structural advantages.

## 2. Electrode Material

### 2.1. Carbon Materials

Carbon materials are mainly used as the electrode materials for Type I supercapacitors and were the earliest electrode materials to be researched and applied. As shown in Table 1, Gamby et al. [27] conducted the first electrochemical characteristic tests on various activated carbons from the PICA Company and observed that the highest specific capacitance was 125 F·g^−1^. Beck et al. [28] prepared industrial carbon black (CB) electrodes with polytetrafluoroethylene (PTFE) as a binder in 12 M H_2_SO_4_; the highest capacitance measured was 250 F·g^−1^. Carbon aerogel is a lightweight porous carbon material with a three-dimensional nanonetwork structure. It has a large specific surface area, low density, high porosity, adjustable pore size distribution, and good electrical conductivity. Fischer et al. [29] obtained carbon aerogel with a density of 800 kg·cm^−3^ via pyrolysis from resorcinol formaldehyde aerogel; the highest capacity measured was 46 F·cm^−3^. Li et al. [30] synthesized mixed carbon aerogels by blending cresol, catechol, and formaldehyde, with a high specific capacitance of 77 F·cm^−3^ (104 F·g^−1^). The good porous structure of carbon aerogels results in their high specific capacitance. Carbon fiber is an inorganic polymer fiber with a carbon content higher than 90%. It has the characteristics of low density, light weight, high strength, and high elastic modulus.

Carbon nanotubes (CNTs) appear as a seamless centrally controlled nanoscale coaxial cylinder rolled by sheet-structured graphite, with fullerene hemispheres sealed at both ends, divided into single-walled carbon nanotubes (SWCNTs) and multi-walled carbon nanotubes (MWCNTs). Compared with the best activated carbon, MWCNTs have available mesoporous structure and the value of their specific capacitance varies up to 135 F·g^−1^ [31]. Liu et al. [32] deposited CNTs on a platinum electrode for the first time, resulting in a specific capacitance of 283 F·g^−1^, which was twice that of an active carbon electrode under the same conditions. Pan et al. [33] reported a tube-to-tube multi-walled carbon nanotube with a specific capacitance of 135 F·g^−1^ consisting of outer nanotubes with an average outer diameter of 50 nm and internal nanotubes with diameters of 3–10 nm. Yang et al. [34] introduced hierarchical graphene sheet (GS)-CNTs with a specific capacitance of 326.5 F·g^−1^. The stacking of GS can be efficaciously impeded by inserting an appropriate quantity of CNTs as nanospacers and enlarging the space between GS sheets, leading to a highly porous nanostructure.

Because of the unique porous structure of carbon materials, they have a larger specific surface area, which facilitates the mass diffusion and transport of electrolytes, providing more ion contact sites and increasing the capacitance. Hollow core, mesoporous shell carbon nanospheres (HCMSs) with a high surface area of 1704 m^2^·g^−1^ exhibited a high specific capacity of 251 F·g^−1^ at 50 mV·s^−1^ in 2 M H_2_SO_4_ [35]. Onion-like carbon (OLC) [36] and nonporous carbons (NPCs) with a metal–organic framework (MOF) as a template exhibited excellent mesoporous characteristics [37]. Zhao et al. [38] fabricated sulfur-containing mesoporous carbons via one-pot aqueous self-assembly with different chemical states of sulfur in tunable amounts. The addition of inorganic elements such as O, N, S, and P improved the electrochemical performance.

### 2.2. Conductive Polymers

Conductive polymers have caused a research boom. Shirakawa et al. [39] first prepared iodine-doped polyacetylene with a conductivity of up to 10^3^ S·cm^−1^. Conductive polymers have the characteristics of high electrical conductivity, large specific surface area, and light specific gravity, as well as good flexibility, low production cost, and high energy efficiency; therefore, they can be used for rechargeable and secondary batteries and electrode materials [40]. Arbizzian et al. [41] synthesized three supercapacitors: a symmetric supercapacitor based on p-doped poly(pyrrole), an asymmetric supercapacitor based on both p-doped poly(pyrrole) and poly(3-methylthiophene), and a symmetric supercapacitor based on p- and n-doped poly(dithieno[3,4-b:3′,4′-d] thiophene) with a high working potential. In Table 2, Ferraris et al. [42] studied various poly 3-(phenylthiophene) derivatives as electrode materials to assemble supercapacitors. The capacitor based on 3-(3, 4 difluorophenyl) thiophene (MPFPT) was particularly promising because of its high capacity and excellent charge/discharge performance. Fusalba et al. [43] synthesized poly(cyclopenta [2,1-b;3,4-b′] dithiophen−4-one) (PCDT) with an open and porous structure. For both the p-type and n-type doped states, the material exhibited a low-frequency capacitance of approximately 70 F·g^−1^.

However, conductive polymers also have shortcomings, such as poor mechanical properties and long-term cycle stability. To overcome these shortcomings and enhance their performance, the optimization of the structure and shape or the mixture of conductive polymers with other carbon materials are important routes. Gouérec et al. [44] showed that the high performance of polyacrylonitrile (PAN) microcellular foam thin films deposited on carbon fibers is related to their high surface area and micropore distribution. Pyrrole (Py) on the surface of a porous graphite fiber matrix resulted in a specific capacitance of 400 F·g^−1^ [45]. Polypyrrole (PPy) on MWCNT membranes exhibits high redox activity, resulting in high specific capacitance [46]. Dubal et al. [47] observed that multilayer PPy nanosheets exhibited a specific capacitance of 586 F·g^−1^ at a scan rate of 2 mV·s^−1^, with a Brunauer–Emmett–Teller (BET) surface area of 37.1 m^2^·g^−1^, which is higher than that of PPy nanoribbons and nanobricks. Jyothibasu et al. [48] prepared PPy tubes using one-step in situ chemical oxidative polymerization with curcumin as a template, which was combined with functionalized carbon nanotubes (f-CNTs) as electrode materials. The electrodes exhibited morphological uniformity, a favorable hierarchical porous structure, a large surface area, and excellent electrochemical properties, including outstanding cycling stability (retention of 118.18% of the initial capacitance after 12,500 charge/discharge cycles).

### 2.3. Metal Compounds

As electrode materials, metal compounds exhibit excellent pseudocapacitance behavior (superior to that of carbon materials), owing to their rapid reversible redox reactions. RuO_2_ is the earliest metal compound used as an electrode material for supercapacitors. Amorphous RuO_2_·xH_2_O is conducive to the diffusion of electrolyte ions in its body phase. The redox reaction not only occurs on the surface of the electrode, but also inside, which is helpful for improving the utilization rate and achieving a high specific capacitance. Compared with RuO_2_·xH_2_O powder [50,51] (Table 3), RuO_2_·xH_2_O thin film is more uniform and complete and has a higher specific capacitance. Scholars have also discovered that the introduction of other metals is helpful for increasing the capacitance because of the synergistic effect between the metals [52,53]. However, the poor conductivity, high cost, and toxicity of RuO_2_ prevents its use in large-scale commercial applications. At this time, relatively inexpensive transition-metal compounds were also shown to possess good pseudocapacitor performance, which was widely valued.

Conway [54] proposed that inexpensive transition-metal oxides have pseudocapacitive behavior, and they were considered the most promising energy storage materials. Prasad et al. [55] prepared nanorod-shaped three-dimensional In_2_O_3_ with high specific capacitance and high power density that exhibited good electrical performance similar to that of the expensive RuO_2_. Nam et al. [56] reported the largest specific capacitance of NiO obtained by electrochemical precipitation with heat treatment at 300 °C; this material has defect properties that can improve its electrochemical properties. In addition to transition-metal oxides [57,58,59,60,61], sulfides, phosphides, and selenides also exhibit pseudocapacitive behavior. These elements’ atoms have larger ionic radii and are more prone to transition transfer and discrete diffusion. To some extent, these materials exhibit improved electrochemical performance. Sulfur atoms have a lower electronegativity than oxygen atoms, and sulfides have a faster redox reaction rate and higher electronic conductivity [62]. Qian et al. [63] prepared CuS nanotubes with a high specific capacitance of 2393 F·g^−1^ at a scan rate of 10 mV·s^−1^. This was the first time that a novel redox-active alkaline electrolyte (polysulfide electrolyte) was used to improve the specific capacitance. A multilayer M-MoS_2_-H_2_O system was first investigated; nanochannels between layers with a distance of approximately 1.18 nm increased the space for ion diffusion and extended the surface area for adsorption [64].

Nano-size compounds with high specific surface area have more active sites and can fully contact the electrolyte, which is beneficial for increasing the specific capacitance [65,66,67,68,69,70]. For the first time, Ramasamy et al. [71] developed a simple colloidal method for the synthesis of CuSbSe_x_S_2−x_ by replacing S with Se; the materials exhibited excellent cyclic stability with promising specific capacitance values. Se atoms can be used to adjust the width of the interlayer gap between layers, which is beneficial for ion diffusion. Lin et al. [72] synthesized coral-like LiFePO_4_ particles through a facile chemical etching method with rugby-like LiFePO_4_ particles as precursors. Priyadharsin et al. [73] prepared γ-KCoPO_4_ nanocrystals as supercapacitor electrodes in an aqueous electrolyte for the first time, resulting in a specific capacitance of 309 C·g^−1^ at 1 mV·s^−1^ in 1 M KOH. Multi-metal compounds exhibit improved electrochemical performance because, in addition to the synergistic pictograms between different components, it is also easy to construct various morphologies with structural advantages [68,69,70,72,74,75].

In general, transition metals are low in cost, easy to obtain, abundant, and exhibit excellent pseudocapacitance performance. Combining transition metals with other materials to construct porous three-dimensional nanostructures with high specific surface area results in electrode materials with high specific capacitance, good energy and power density, and good stability.

### 2.4. Composite Materials

Composite materials are more suitable for the development of supercapacitor electrode materials because of their improved electrochemical performance relative to that of single-component materials, resulting from the synergy between the components. It is easier to build porous nanostructures with high specific surface area, which can provide more electroactive sites and enable fast electron transmission and enhanced structural stability. As shown in Table 4, many attempts have been made to study composite materials with carbon materials [76,77,78,79]. Electrochemical measurements showed that the pseudocapacitance behavior of RuO_2_ increases the specific capacitance of pure carbon materials. Transition metals have the potential to replace precious metal oxides in supercapacitor electrodes. Scientists are also committed to developing a low-cost, high-performance material. Composite materials of transition-metal compounds and various carbon materials exhibit better performance than pure carbon materials [80,81,82,83,84,85]. It is worth emphasizing the use of CNTs, graphene, carbon fiber, and molecular sieves. These materials have high specific surface area and mesoporous structures, which are very conducive to the construction of composite materials with structural advantages [86]. Co(OH)_2_/ultrastable Y molecular sieve composites [87], NiO/CNT composites [88], Co_3_O_4_ nanowire/three-dimensional graphene foam [89], Ni_3_S_2_ nanoparticles/MWCNTs [90], and α-Ni(OH)_2_-GO composites [91] all exhibit high specific capacitance, with the highest capacitance reached being 1760.72 F·g^−1^ [91].

Binary and even multi-component metal compound composite materials exhibit superior performance because of the synergistic effect between metals and between different components. BiMnO_5_/MWCNT composites were used as a new active material for positive electrodes of supercapacitors, with a maximum energy density of 9.0 Wh·kg^−1^ and maximum power density of 2.5 kW·kg^−1^ for an asymmetric device [92]. A Co–Al layered double hydroxide nanosheet (Co–Al LDH–NS)/GO film composite [93] exhibited a specific capacitance of 1200 F·g^−1^ and a long cyclic life owing to the well-organized layered structure, which was beneficial to efficient electron transport. A CoMoO_4_/G composite exhibited low electrochemical resistance, good rate capability, and good cycle life, and possessed a higher specific surface area and electroactive area than pure CoMoO_4_, promoting the acquisition of OH^−^ and fast charge transfer [94]. For N-doped graphene, the N atoms inserted in the carbon lattice played a significant role in improving the capacitance and stability of the electrode material because of the local structural deformation around N atoms as well as the Coulomb effect [95,96].

Co-incorporated NiV_2_O_6_/Ni(HCO_3_) nanosheet arrays directly grown on Ni foam possessed long-term durability, with 106.2% retention of the initial capacity after 10,000 charging/discharging cycles at 100 mA·cm^−2^. The electrons transferred from the V center to the Ni active sites because of the synergistic contribution of the individual components, making the structure more stable [97]. Composite materials with multiple metal components exhibit high specific capacitance, good rate capability, and improved cycling stability, profiting from the high specific surface area, plentiful surface-active sites, good interfacial conductivity, and porous structure [98,99,100,101].

Building composite materials with a well-layered core–shell structure is conducive to enhancing electrochemical performance. Because of the synergistic contribution between the core and shell, the high specific surface area provides more electroactive sites for Faradaic redox reactions and increases ion and electron diffusion; thus, composite materials with core–shell structures exhibit satisfactory electrochemical performance [102,103,104].

## 3. Materials Based on CC

**Figure 2:**
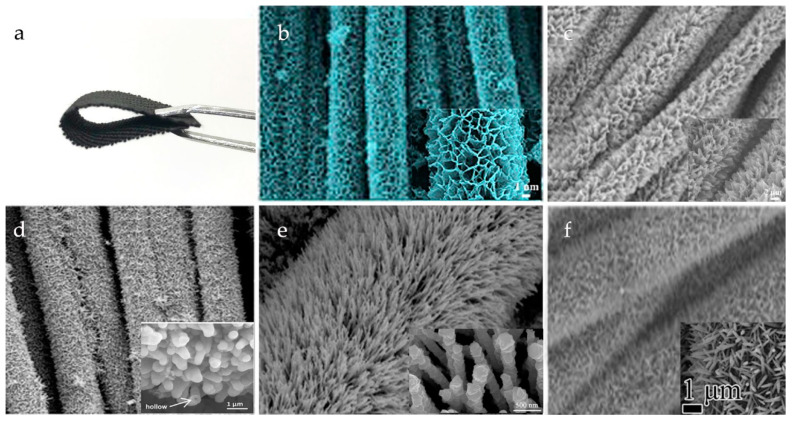
(**a**) Digital photograph of commercial CC with area of 1 × 4 cm^2^; (**b**–**f**) SEM images of several compounds with different structures grown on CC [107,108,109,110,111]. Adapted from [108,109,110,111], with permission from Elsevier, 2020.

A summary of the electrochemical performance of electrode materials based on CC is provided in Table 5. Rowlands et al. [105] concluded that CC as an electrode exhibits a specific capacitance of 35 F·g^−1^. It is worth mentioning that Dai et al. [106] developed hierarchical porous hollow N-doped CC as an electrode material for organic-electrolyte supercapacitors, overcoming the shortcomings of pure CC with superior stability (98% capacitance retention over 20,000 cycles). Compared with electrode materials without a substrate, the advantages of materials based on CC are reflected in the following points. The compound can grow in situ on CC and extend outward, and the growth is more uniform, resulting in a larger surface area and porous structure, shortening the ion-diffusion channel, accelerating the transmission path of electrons and ions, accelerating the Faraday capacitance reaction, and enhancing the electrolysis of liquid and electrode materials. CC also exhibits good flexibility and plasticity, as observed in the digital photograph of commercial CC, with an area of 1 × 4 cm^2^ (Figure 2a). As shown in Figure 2b, Liu et al. [107] synthesized uniform honeycomb-like NiCo_2_S_4_ nanosheets grown on CC with an excellent capacitance up to 1638 F·g^−1^ at 1 A·g^−1^. Graphene quantum dots on carbon cloth can somewhat improve specific capacitance over carbon cloth and maintain perfect flexibility, but its performance is far from adequate [10]. Zhao et al. [108] prepared a metal–organic framework using an etching/ion-exchange method, as shown in Figure 2c; the obtained ultra-thin nanosheet arrays with a mesoporous structure exhibited good conductivity with abundant active sites, resulting in enhanced electrochemical performance and a high specific capacitance of 2392 F·g^−1^ at a current density of 1 A·g^−1^. A Ni–Co–S/ACC//AC asymmetrical flexible supercapacitor exhibited good cycling stability (82% retention after 10,000 cycles). The tortoise-shell-like structure α-Fe_2_O_3_/C nanoarray on CC is shown in Figure 2d. The hollow porous structure of the α-Fe_2_O_3_/C nanoarray, carbon nanoparticles, and numerous mesopores between the α-Fe_2_O_3_ nanocrystals and carbon nanoparticles all improved the electrochemical performance [109]. The hierarchical NiCo_2_S_4_@NiCo_x_S_y_ core/shell nanoarrays grown on CC shown in Figure 2e and hierarchical tectorum-like α-Fe_2_O_3_/PPy nanoarrays grown on CC shown in Figure 2f exhibited areal capacitances as high as 3.9 F·cm^−2^ at 1 mA·cm^−2^ and 382.4 mF·cm^−2^ at 0.5 mA·cm^−2^, respectively [110,111]. Besides above advantages, electrode materials based on CC have more active sites, increasing the energy storage and capacitance [112,113,114].

The use of base materials such as CC for constructing nano-sized forms is an effective way to improve the electrochemical performance of electrode materials by shortening the length of the ion-diffusion channel [115,116,117,118,119,120]. Horng et al. [121] prepared nano-sized polyaniline nanowires on CC (PANI-NWs/CC) via an electrochemical method; the materials exhibited specific capacitance up to 1220 F·g^−1^ while overcoming the cycle degradation issues caused by mechanical issues. The one-dimensional structure of the metal nanowires as the supporting framework of the fixing material effectively produced a porous structure and improved the performance and stability of the materials with good conductivity and fast charge transport [122,123].

The preparation methods of electrode materials consist of electrochemical deposition, chemical deposition, dip dyeing, and hydrothermal methods. Compounds obtained under hydrothermal conditions can overcome the hard agglomeration of certain high-temperature preparation methods. The characteristics of hydrothermally produced materials include high particle purity, small size (nano-level), good dispersion, uniform distribution, no agglomeration, good controllability, low production cost, and environmentally friendly reaction conditions and atmosphere. In addition, from the perspective of environmental protection, the hydrothermal method is a mild, green, low-cost, and easy-to-operate preparation method. It has become an important choice for the preparation of electrode materials after ongoing improvement.

**Figure 3:**
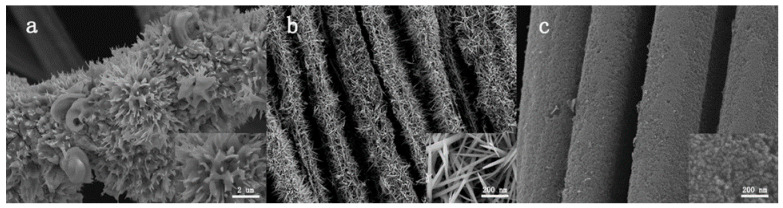
SEM images of (**a**) sea-urchin-like Ni–Co–S compound on CC, (**b**) Ni–Co–S nano-needle on CC, and (**c**) Ni–Co–Fe–S nanoparticles on CC.

Figure 3 presents SEM images of multiple metal sulfides based on CC prepared via the hydrothermal method; a sea-urchin-like Ni–Co–S compound on CC with a high surface area and many active sites is displayed in Figure 3a. However, it can be observed that the high surface energy results in local agglomeration. After improving the experimental conditions, a good structure of nano-needle nickel cobalt sulfide growing on CC with excellent electrochemical performance was obtained (Figure 3b). In Figure 3c, a small amount of Fe element was added under the same experimental atmosphere as Figure 3b; the Ni–Co–Fe–S nanoparticles are uniformly distributed on the carbon cloth with scarce agglomeration of large particles.

## 4. Main Existing Issues, Corresponding Solutions, and Future Work

Although electrode materials exhibit great potential due to low cost, high performance, and good flexibility, many unanswered questions remain. Among the electrode materials, carbon materials with multidimensional pore characteristics, graphene, and atom-doped carbon materials have developed dramatically. In the process of basic research on electrode materials, various advanced design concepts have driven the rapid development of related fields and industries.

However, low load is completely inadequate to meet the needs of commercial electrodes. Therefore, increasing the active material load of the electrode and further exploring and optimizing its electrochemical performance will provide a reference and guide for its future practical application.

Advanced technology was used to reveal the phase evolution law of materials and the structure-effect relationship between structure and properties, so as to guide the design and construction of electrode materials. We can see the future trend of electrode materials from bionic carbon materials, but the huge technical challenges are still difficult problems.

As a substrate growth material to construct a variety of three-dimensional structural composite materials, especially with the redox properties of transition metals to build porous structures with high specific surface area, the performance of composite material-based carbon materials are surprising.

For three-dimensional composite materials with a controllable nanostructure based on CC, the most important thing is that they provide more active sites and exhibit excellent electrochemical properties [124,125,126,127]. We can see that metal composites exhibit oxidative performance, which is the advantage that other materials cannot achieve. However, the controllability and stability of the structure are large problems. When the growth dimension reaches nano-level, especially, the structure has a larger surface area, there are more active sites, and at the same time the surface energy is greater, agglomeration is easier, and the structure can be more easily damaged. The hydrothermal method not only does not destroy the structure and performance of the CC, but also provides a mild environment for the growth of composite material based on CC, and three-dimensional nano-structures exhibit more possibilities and potential.

## 5. Conclusions

Supercapacitors have the advantages of high power density, fast charge and discharge rates, long cycle life, high safety, and reliability as a new generation of green energy materials to replace traditional energy materials. The hydrothermal method has the characteristics of simplicity, environmental friendliness, and uniform growth of the load. Nanostructures can be grown directly on CC via a hydrothermal method, including nano-needles, tree-like and nano-flower shapes, ultra-thin nanosheets, and tectorum-like shapes, which have high surface areas and porous structures, shorten the transmission channels of ions and electrons, and increase the active sites for the Faraday capacitance reactions. The synergistic effect between the components also effectively improves their specific capacitance, cycle stability, and rate capability. In addition, flexible supercapacitors assembled with electrode materials based on CC have great advantages for the energy supply for wearable devices.

## Figures and Tables

**Table 1:** Carbon materials for supercapacitors.

Material	The Highest Capacities	Specific Surface Area	Specific Power Densities	Specific Energy Densities	Specific Capacitance Retention	Ref.
Activated carbon	125 F·g^−1^	-	-	-	-	[27]
CBs	250 F·g^−1^	28–1690 m^2^·g^−1^	-	-	-	[28]
Carbon aerogels	46 F·cm^−3^	-	-	-	-	[29]
Carbon aerogels	77 F·cm^−3^ (104 F·g^−1^)	-	-	-	-	[30]
MWCNTs	135 F·g^−1^	470 m^2^·g^−1^	-	-	-	[31]
SWCNTs	283 F·g^−1^	-	-	-	-	[32]
Tubes-in-tube CNTs	315 F·g^−1^	-	-	-	-	[33]
GS-CNTs	326.5 F·g^−1^	-	78.29 kW·kg^−1^	21.74 Wh·kg^−1^	-	[34]
HCMSs	251 F·g^−1^	-	-	-	98% (after 200 cycles)	[35]
OLC	-	-	195.0 W·cm^−3^	2.9 mWh·cm^−3^	-	[36]
NPCs	222 F·g^−1^	-	-	-	-	[37]
S-carbon	-	-	-	-	-	[38]

**Table 2:** Properties of conductive polymers for supercapacitors prepared by various methods.

Materials	Method	Specific Capacitance	Specific Power Densities	Specific Energy Densities	Ref.
Poly 3-(Phenylthiophene) derivatives	Electrochemical polymerization	-	5 kW·kg^−1^	50 Wh·kg^−1^	[42]
PCDT	Electrochemical polymerization	-	1 kW·kg^−1^	6 Wh·kg^−1^	[43]
PAN	Deposition	-	-	-	[44]
Py	Chemical polymerization	400 F·g^−1^	-	-	[45]
PPy	Electrochemical deposition	427 F·g^−1^	-	-	[46]
PPy	Electro polymerization	586 F·g^−1^	-	-	[47]
PPy	In-situ chemical oxidative polymerization	2732 mF·cm^−2^	129.35 mW·cm^−2^	242.84 µWh·cm^−2^	[48]
(E)-α-cyanoethylene thiophene derivatives	Electrochemical polymerization	-	1.6 kW·kg^−1^	8.6 Wh·kg^−1^	[49]

**Table 3:** Properties of metal compounds for supercapacitors prepared by various methods.

Materials	Method	Specific Capacitance	Specific Power Density	Specific Energy Density	Specific Capacitance Retention	Ref.
RuO_2_·xH_2_O powder	Sol-gel process	720 F·g^−1^	-	26.7 Wh·kg^−1^	-	[50]
RuO_2_ films	Electrodeposition	1190 F·g^−1^	-	-	-	[51]
Ru_0.6_Sn_0.4_O_2_·nH_2_O	Hydrothermal	830 F·g^−1^	-	-	-	[53]
RuO_2_-VO_2_ solid solution	Polymerizable-complex	1210 F·g^−1^	-	-	-	[54]
In_2_O_3_	Electrochemical deposition	190 F·g^−1^	-	-	-	[55]
NiO film	Electrochemical precipitation	-	-	-	-	[56]
NiO nanosheet	Hydrothermal	989 F g−1	-	49.45 Wh·kg^−1^	97% (after 1000 cycles)	[57]
cobalt oxide	Deposition	165 F·g^−1^	-	-	-	[58]
CuO multilayer nanosheets	CBD	43 F·g^−1^	-	-	-	[59]
MnO_2_	-	398 F·g^−1^	-	-	-	[60]
Mn_3_O_4_ thin films	SILAR	314 F·g^−1^	-	-	-	[61]
CoS nanowires	Biomolecule-assisted hydrothermal	508 F·g^−1^	-	-	-	[62]
CuS nanotubes	-	2393 F·g^−1^	-	-	-	[63]
M-MoS_2_-H_2_O system	Hydrothermal	380 F·g^−1^	-	-	-	[64]
Ni(OH)_2_	Hydrothermal	1715 F·g^−1^	-	-	-	[65]
α-Co(OH)_2_ nanowire arrays (NWAs)	Hydrothermal	642.5 F·g^−1^	-	-	-	[66]
FeVO_4_ nanoparticles	Co-precipitation	972 F·g^−1^	1326 kW·kg^−1^	21 Wh·kg^−1^	-	[67]
NiCo_2_O_4_ NSs@HMRAs	Electrodeposition	678 F·g^−1^	-	15.42 Wh·kg^−1^	96.06% (after 1500 cycles)	[68]
CoNi_2_S_4_	Hydrothermal	2906 F·g^−1^	409 W·kg^−1^	33.9 Wh·kg^−1^	-	[69]
Sn-Co binary oxide nanosheets	Hydrothermal	937.4 F·g^−1^	-	-	97.5% (after 20,000 cycles)	[70]
CuSbSe_2_	Colloidal	-	-	-	99.5% (after 200 cycles)	[71]
Coral-Like LiFePO_4_ Particles	Chemical etching	359 F·g^−1^	-	-	82.3% (after 2000 cycles)	[72]
γ-KCoPO4	Sol-gel	222 C·g^−1^	1.6 kW·kg^−1^	28 Wh·kg^−1^	-	[73]
Mn-Co-Fe HNPs	Electrodeposition	1200 F·g^−1^	1125 W·kg^−1^	11.4 Wh·kg^−1^	96% (after 4000 cycles)	[74]
Co-Mo-O-S porous microspheres	Hydrothermal	1134 F·g^−1^	-	67.6 Wh·kg^−1^	-	[75]

**Table 4:** Properties of composite materials for supercapacitors prepared by various methods.

Materials	Method	Specific Capacitance Density	Specific Power Density	Specific Energy Density	Specific Capacitance Retention	Ref.
Carbon-ruthenium xerogels	Sol-gel	256 F·g^−1^	-	-	Almost 100% (> 2000 cycles)	[76]
RuO_2_/CNT	-	340 F·g^−1^	-	-	-	[77]
RuO_2_·xH_2_O/CB	Novel incipient wetness	647 F·g^−1^	-	-	-	[78]
Co, Mn, Cu, Fe, Zn-doped carbon aerogels	Impregnation	100 F·g^−1^ (Co) 107 F·g^−1^(Mn)	-	-	-	[79]
MoO_3_/AC	Impregnation	177 F·g^−1^	-	-	94% (after 20,000 cycles)	[80]
ITO/AC	Reverse precipitation	-	-	-	-	[81]
WO_3_/CA carbon aerogel	Immersion-calcination	700 F·g^−1^	-	-	95% (after 4000 cycles)	[82]
Graphene-MnO_2_	Self-limiting deposition	310 F·g^−1^	-	-	-	[83]
Mesh-like Fe_2_O_3_/C	Template free greener	315 F·g^−1^	-	37 Wh·kg^−1^	88.9% (after 1500 cycles)	[84]
CuO-NC	Hard templating	300 F·g^−1^	-	-	91% (after 1000 cycles)	[85]
Co(OH)_2/_USY	Chemical precipitation	958 F·g^−1^	-	-	-	[86]
NiO/CNT	Hydrothermal	1329 F·g^−1^	-	-	-	[87]
Graphene/Co_3_O_4_ nanowire	Hydrothermal	1100 F·g^−1^	-	-	-	[88]
Graphene sheets/Ag_2_S	Solvothermal	1063 F·g^−1^	-	-	-	[89]
Ni_3_S_2_/MWCNT	Hydrothermal	800 F·g^−1^	798 W·kg^−1^	19.8 Wh·kg^−1^	90% (after 5000 cycles)	[90]
α-Ni(OH)_2_-GO	Hydrothermal	1760.7 F·g^−1^	-	-	-	[91]
BiMn_2_O_5_-MWCNT	Hydrothermal	540 F·g^−1^	3.6 kW kg^−1^	13 Wh kg^−1^	-	[92]
Co-Al LDH-NS/GO	LBL	880 F·g^−1^	-	-	-	[93]
CoMoO_4_/graphene	Hydrothermal	394.5 F·g^−1^	197.2 W·kg^−1^	54.8 Wh·kg^−1^	-	[94]
NG-NiMnO_3_	Hydrothermal	750.2 F·g^−1^			-	[95]
Co_3_S_4_-NG	Hydrothermal	2427 F·g^−1^			-	[96]
NiV_2_O_6_/Ni(HCO_3_)_2_ nanoflake arrays	Hydrothermal	7.94 F·cm^−2^	4.983 mW·cm^−2^	0.415 mWh·cm^−2^	106.2% (after 10,000 cycles)	[97]
Mn/PbO_x_ Mn/NiO_x_	Chemical reduction	185 F·g^−1^ 210 F·g^−1^			-	[98]
Co_3_O_4_/Ni(OH)_2_O	Electrochemical deposition	1144 F·g^−1^			-	[99]
NiMoO_4_/CoMoO_4_ nanorods	Hydrothermal	1164 F·g^−1^	3750 W·kg^−1^	17.5 Wh·kg^−1^	87.5% (after 3000 cycles)	[100]
Ag QDs/NiMoO_4_	Dipping and drying	3342.7 F·g^−1^	212.5 kW·kg^−1^	48.5 Wh·kg^−1^	-	[101]
Ppy@NiCo_2_S_4_ core-shell heterostructure	Hydrothermal	908.1 F·g^−1^	160 W·kg^−1^	50.82 Wh·kg^−1^	126.6% (after 2000 cycle)	[102]
Co@Co_3_O_4_ core-shell 3DN	Surface oxidating	1049 F·g^−1^	-	-	-	[103]
Core-shell hollow CoMoS_4_@Ni-Co-S nanotubes	Hydrothermal Electrodeposition	2208.5 F·g^−1^	800 W·kg^−1^	49.1 Wh·kg^−1^	90.3% (after 10,000 cycles)	[104]

**Table 5:** Properties of materials based on CC for supercapacitors prepared by various methods.

Materials	Method	Specific Capacitance Density	Specific Power Density	Specific Energy Density	Specific Capacitance Retention	Ref.
CC	Electrochemical anodization	35 F·g^−1^	-	-	-	[105]
N-doped activated CC	One-step etching & doping (E&D)	215.9 F·g^−1^	-	-	98% (20,000 cycles)	[106]
Graphene Quantum Dots (GQD)/CC	Peroxide-assisted hydrothermal	70 mF·cm^−2^				[10]
NiCo_2_S_4_/CC	Hydrothermal	1638 F·g^−1^	799.6 W·kg^−1^	25.2 Wh·kg^−1^	-	[107]
Ni-Co-S/ACC	Hydrothermal	2392 F·g^−1^	800.2 W·kg^−1^	30.1 Wh·kg^−1^	82% (10,000 cycles)	[108]
α-Fe_2_O_3_/C nanoarrays on CC	Hydrothermal	391.8 F·g^−1^	-	-	91.8% (4000 cycles)	[109]
NiCo_2_S_4_@NiCoxSy on CC	Hydrothermal	3.9 F·cm^−2^	-	-	-	[110]
α-Fe_2_O_3_/PPy nanoarrays on CC	Hydrothermal and in situ vapor-phase polymerization	382.4 mF·cm^−2^	-	-	-	[111]
Hierarchical Co(OH)_2_@ NiMoS_4_ on CC	Hydrothermal	2229 F·g^−1^	1000 W·kg^−1^	159.5 Wh·kg^−1^	100% (5000 cycles)	[112]
ZIF−67 on CC	Dipping	829 F·g^−1^	-	-	103% (15,000 cycles)	[113]
ROCC@PDAA on CC	In-situ chemical oxidation polymerization	81.9 F·g^−1^	-	-	159% (20,000 cycles)	[114]
NiO nanoflake arrays on CC	CBD	660 F·g^−1^	-	-	82% (4000 cycles)	[115]
MoO_3_ film on CC	Electrodeposition	835 F·g^−1^	1000 W·kg^−1^	78 Wh·kg^−1^	98% (8000 cycles)	[116]
MoS_2_ nanospheres on CC	Hydrothermal	368 F·g^−1^	128 W·kg^−1^	5.42 Wh·kg^−1^	96.5% (after 5000 cycles)	[117]
Ni(OH)_2_ on CC Co(OH)xCO_3_ on CC	Hydrothermal	789 F·g^−1^ 550 F·g^−1^	1.4 kW·kg^−1^	41.1 Wh·kg^−1^ 33.48 Wh·kg^−1^	98%; 97.6% (after 5000 cycles)	[118]
P-doped NiCo_2_S_4_ nanotube arrays on CC	Hydrothermal	8.03 F·cm^−2^	750 W·kg^−1^	42.1 Wh·kg^−1^	87.5% (5000 cycles)	[119]
NCLP@ NiMn-LDH on CC	Hydrothermal	2318 F·g^−1^	750 W·kg^−1^	42.2 W h kg^−1^	-	[120]
PANI-NWs/CC	Electrochemical polymerization	1079 F·g^−1^	12.1 kW·kg^−1^	100.9 Wh·kg^−1^	-	[121]
NC LDH NSs@Ag@CC	Electrochemical deposition	1133.3 mF·cm^−2^	-	-	80.47% (2000 cycles)	[122]
Ni-Co LDH Nanoflakes–ZnO nanowires hybrid array on CC	Hydrothermal	927 F·g^−1^	46.15 kW·kg^−1^	45.55 Wh·kg^−1^	96.02% (3000 cycles)	[123]

## Data Availability

Not applicable.
